# Supplementary material for: Identification and characterisation of Mansonella perstans in the Volta Region of Ghana
Source: PLoS One. 2024 Jun 7;19(6):e0295089. doi: 10.1371/journal.pone.0295089 (PMC11161070; doi:10.1371/journal.pone.0295089)
Supplement: S1 Table — The number of individuals positive for M. perstans are shown. (PDF) [file pone.0295089.s001.pdf]

District	Community	Number positive	Latitude	Longitude
Adaklu	Ablornu	3	6.35	0.64
Adaklu	Afeyeame	7	6.39	0.64
Adaklu	Anfoe	1	6.46	0.64
Hohoe	Abledzie	23	7.07	0.51
Hohoe	Akpatanu	4	7.17	0.46
Hohoe	Dzenana	1	7.11	0.49
