# Supplementary material for: Identification and characterisation of Mansonella perstans in the Volta Region of Ghana
Source: PLoS One. 2024 Jun 7;19(6):e0295089. doi: 10.1371/journal.pone.0295089 (PMC11161070; doi:10.1371/journal.pone.0295089)
Supplement: S2 Table — Those that were submitted for Sanger sequencing are indicated with *. (PDF) [file pone.0295089.s002.pdf]

**Community**	**Participant ID**	**Assession No.**	**Total mf count**	**mf/ml**	**ITS-1**
Dzenana	HH/DZN 010	OR488627	1	16.7	1 *
Abledzie	HH/ABZ 065	OR488628	2	33.3	1 *
Abledzie	HH/ABZ 063	OR488629	741	12350.0	1 *
Abledzie	HH/ABZ 009	OR488630	9	150.0	1 *
Afeyeame	AD/AFY 079	OR488631	5	83.3	1 *
Akpatanu	HH/AKA 029	OR488632	331	5516.7	1 *
Abledzie	HH/ABZ 072	OR488633	42	700.0	1 *
Abledzie	HH/ABZ 010	OR488634	37	616.7	1 *
Ablornu	AD/AAB 020	OR488635	4	66.7	1 *
Abledzie	HH/ABZ 016	OR488636	71	1183.3	1 *
Ablornu	AD/AAB 030	OR488637	14	233.3	1 *
Afeyeame	AD/AFY 054	OR488638	1	16.7	1 *
Abledzie	HH/ABZ 015	OR488639	51	850.0	1 *
Abledzie	HH/ABZ 021	OR488640	10	166.7	1 *
Abledzie	HH/ABZ 097	OR488641	48	800.0	1 *
Afeyeame	AD/AFY 004	OR488642	5	83.3	1 *
Abledzie	HH/ABZ 022	OR488643	52	866.7	1 *
Abledzie	HH/ABZ 067	OR488644	2	33.3	1 *
Abledzie	HH/ABZ 088	OR488645	109	1816.7	1 *
Akpatanu	HH/AKA 041	OR488646	43	716.7	1 *
Abledzie	HH/ABZ 020	OR488647	14	233.3	1 *
Abledzie	HH/ABZ 007	OR488648	55	916.7	1 *
Abledzie	HH/ABZ 098	OR488649	42	700.0	1 *
Akpatanu	HH/AKA 040	OR488650	98	1633.3	1 *

Akpatanu	HH/AKA 028	OR488651	6	100.0	1 *
Abledzie	HH/ABZ 003		11	183.3	1 *
Abledzie	HH/ABZ 027		19	316.7	1 *
Abledzie	HH/ABZ 043		198	3300.0	1 *
Abledzie	HH/ABZ 074		11	183.3	1 *
Abledzie	HH/ABZ 013		4	66.7	0
Abledzie	HH/ABZ 040		1	16.7	0
Abledzie	HH/ABZ 062		1	16.7	0
Abledzie	HH/ABZ 069		1	16.7	0
Afeyeame	AD/AFY 006		1	16.7	0
Afeyeame	AD/AFY 024		4	66.7	0
Afeyeame	AD/AFY 026		1	16.7	0
Afeyeame	AD/AFY 070		3	50.0	0
Ablornu	AD/AAB 089		37	616.7	0
Anfoe	AD/ANF 059		1	16.7	0
