# Supplementary material for: Identification and characterisation of Mansonella perstans in the Volta Region of Ghana
Source: PLoS One. 2024 Jun 7;19(6):e0295089. doi: 10.1371/journal.pone.0295089 (PMC11161070; doi:10.1371/journal.pone.0295089)
Supplement: S3 Table — T92+G model was selected to build the tree based on low BIC and AICc values. (PDF) [file pone.0295089.s003.pdf]

Model	#Param	BIC	AICc
T92+G	78	4267.517123	3679.621523
T92	77	4271.068361	3690.698778
T92+I	78	4271.503083	3683.607483
HKY+G	80	4285.80598	3682.859216
HKY	79	4289.324916	3693.903589
HKY+I	80	4289.79159	3686.844826
TN93+G	81	4294.287025	3683.815115
T92+G+I	79	4296.003595	3700.582268
TN93	80	4298.00774	3695.060975
TN93+I	81	4298.315192	3687.843281
HKY+G+I	81	4314.268701	3703.79679
GTR+G	84	4316.620709	3683.575101
GTR	83	4318.907896	3693.386563
GTR+I	84	4320.11899	3687.073381
TN93+G+I	82	4322.986873	3704.990106
JC+G	76	4324.194239	3751.350963
JC	75	4324.566891	3759.250212
JC+I	76	4325.907618	3753.064342
GTR+G+I	85	4331.040315	3690.470721
JC+G+I	77	4333.69955	3753.329967
K2	76	4336.855068	3764.011792
K2+I	77	4339.880374	3759.510791
K2+G	77	4346.913583	3766.544
K2+G+I	78	4372.686321	3784.790721
