# Supplementary figures and images for: Identification and characterisation of Mansonella perstans in the Volta Region of Ghana
Source: PLoS One. 2024 Jun 7;19(6):e0295089. doi: 10.1371/journal.pone.0295089 (PMC11161070; doi:10.1371/journal.pone.0295089)

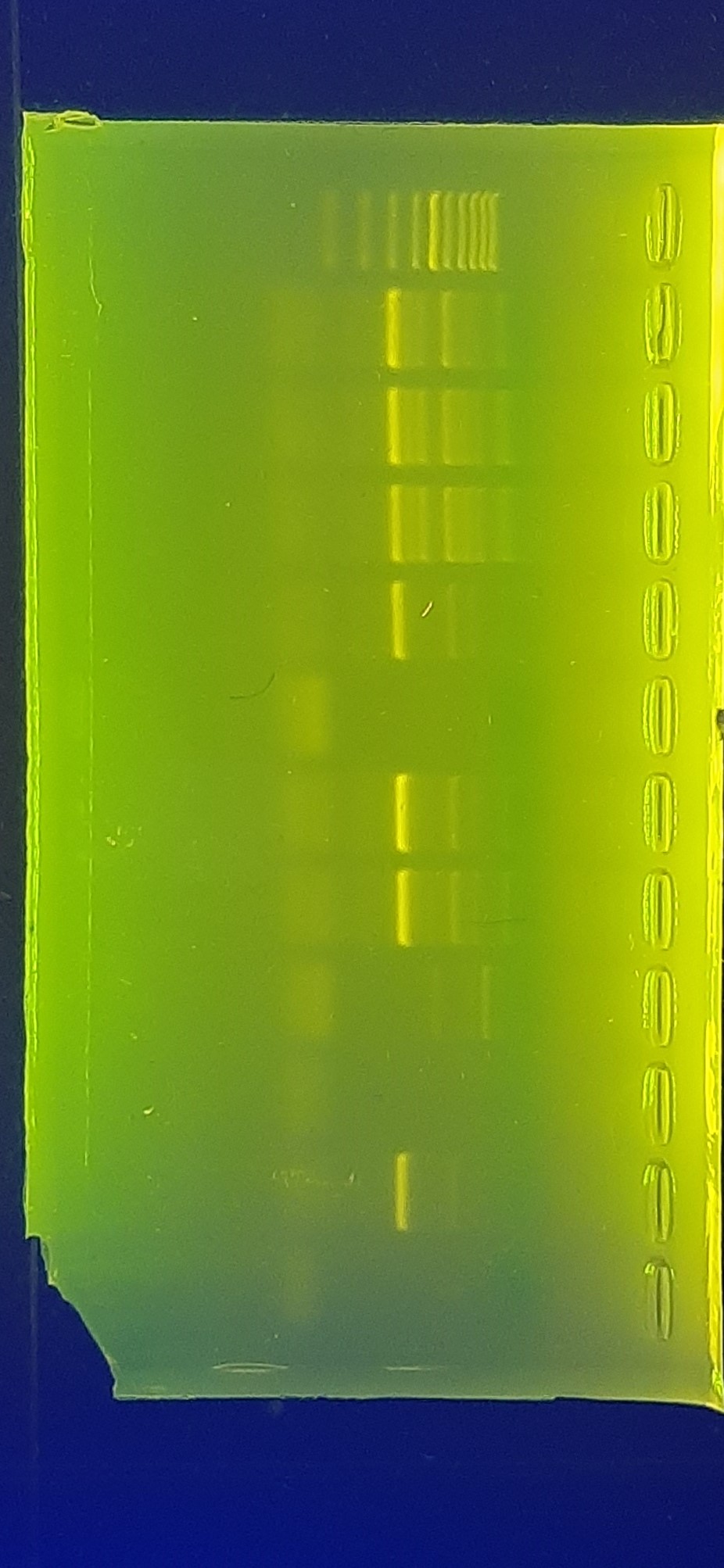

Supplement: S1 Raw Image — (JPG) [file pone.0295089.s004.jpg]
