# Supplementary material for: Identification and characterisation of Mansonella perstans in the Volta Region of Ghana
Source: PLoS One. 2024 Jun 7;19(6):e0295089. doi: 10.1371/journal.pone.0295089 (PMC11161070; doi:10.1371/journal.pone.0295089)
Supplement: Attachment — Submitted filename: Rebuttal Letter.pdf [file pone.0295089.s006.pdf]

April 26, 2024

Editor-in-Chief

PloS One

Dear Editor,

We are grateful for your consideration to publish our work in your reputable journal. Below are the responses to the comments from reviewers and the editor in red ink.

Reviewer #1

1. LINES 43-44: Despite its widespread distribution, it is the least studied among all the filarial infections."

   Response: This has been corrected on line 44.

2. LINES 50-53; Change to: " Prior to sequencing, genomic DNA was extracted and a nested PCR reaction carried out. A total of 30 samples that were positive from the nested PCR with a band size of ~312bp were submitted for Sanger sequencing.

   Response: Sequences has been changed to sequencing on line 54.

3. LINES 71-71: Please delete this line: Some mosquito species including *Aedes* and *Anopheles* have also been implicated (4, 10, 11). This is miss-leading and is not supported by the cited references.

   Response: This line has been deleted.

4. LINE 72-74: "*M. ozzardi* is the major species in Latin America and the Caribbean, while *M. perstans* and *M. streptocerca* are reported in Africa." This requires a reference, I suggest: Portela CS, Mendes de Araújo CP, Moura Sousa P, Gomes Simão CL, Silva de Oliveira JC, Crainey JL.Filarial disease in the Brazilian Amazon and emerging opportunities for treatment and control.Curr Res Parasitol Vector Borne Dis. 2023 Dec 23;5:100168. doi: 10.1016/j.crpvbd.2023.100168. eCollection 2024.

   Response: The sentence has been updated to **"Of the three species, M. ozzardi is the major species in Latin America and the Caribbean, while M. perstans and M. streptocerca are reported in Africa"** and the suggested reference included on lines 69-70.

5. LINE 88: *Wolbachia* is a genus name and thus should always be in italics.

   Response: *Wolbachia* has been italicised on line 89.

6. LINE 94: "In Ghana, Mansonellosis was first reported in the 1991 by Awadzi and colleagues (25)". This line needs to be changed. Mansonellosis infections are not only caused by M. ozzardi and M. perstans they are also caused by M. streptocerca and the first reports of M. streptocerca were made from samples taken in Ghana (see Macfie and Corson 1922). Macfie, J. W. S., & Corson, J. F. (1922). A new species of filarial larva found in the skin of natives in the Gold Coast. Annals of Tropical Medicine & Parasitology, 16(4), 465-471.

Response: The statement has been corrected and now reads "**The first report of mansonellosis was in 1922 where, M. streptocerca was identified in the skin of indigenes from Gold Coast, now Ghana**" and the reference included on lines 95-96.

7. LINES 133-114:) "as well as vaccine uptake (1, 5, 36)." Should it not be efficacy instead of uptake?

   Response: uptake has been replaced with efficacy

9. LINES 152-153:Change to: "A, showing the map of Ghana with the districts where samples were collected."
10. LINES 155-156: This does not make sense, please revise: "shows the Hohoe district (Volta Region) in orange with communities in grey and yellow circles for M. perstans negative and positives respectively that were positive for". Please also always put *M. perstans* in italics.

    Response: Lines have been changed and reads **"A, showing the map of Ghana with the districts where samples were collected. B, shows the East Akyim district (comprising the Abuakwa North and South Districts (Eastern Region)) in green with communities sampled in pale orange circles. C, shows the Hohoe district (Volta Region) in orange. D, shows three districts (Adaklu, Agotime Ziope and Central Tongu Districts in purple, grey and brown respectively) also in the Volta Region. Communities sampled are in circles with yellow and grey indicating sites with *M. perstans* positives and negatives respectively**" on lines 157 - 163.

    M. perstans is now italicised on line 163.

10. LINES 174-176: Change to: "The liquid containing DNA was transferred (whilst ensuring that no particles of the filter paper is pipetted with the sample) to a Zymo-Spin™ IICR Column placed in a Collection Tube."

    Response: the sentence has been modified and now reads "**The liquid containing the DNA was transferred to a Zymo-Spin™ IICR Column placed in a Collection Tube (ensure that no particles of the filter paper is pipetted with the sample)." Lines 178-180.**

11. LINES 212-213: "visualised under blue led light." Should this be UV light?

    Response: The gel was visualised with a blue light transilluminator not the usual UV light using the Dual LED Blue/White Light Transilluminator machine from Bio-Helix. The name of the machine has been indicated on line 216.

12. LINE 260: Change to: Fig 2. Gel electrophoresis photograph showing Nest II PCR products.

    Response: Gel electrogram has been changed to Gel electrophoresis photograph on line 263.

Reviewer #2
Abstract:

1. Change: Lao loa -> Loa loa (line 24)

   Response: Lao has been changed to *Loa* on line 24

2. Suggestion: Occurrence -> Co-occurrence (line 24)

Response: Occurrence has been changed to co-occurrence on line 23

3. Remove: "Hence," (line 25)

   Response: This has been removed. Also, the sentence has been modified to now read **"There are few surveillance programmes for assessing the distribution of mansonellosis, due to the associated mild to no symptoms experienced by infected people"** on lines 24-25.

4. Change: distribution or mansonellosis -> distribution of mansonellosis (line 26)

   Response: "or" has been changed to **"of"** on line 25

5. Change: giemsa -> Giemsa (Capitalize the "G", line 31)

   Response: giemsa has been correct to start with a capital **"G"** on line 30.

6. Clarify: lines 37-38 change to "...supporting the hypothesis that M. perstans was introduced into the Americas during the transatlantic slave trade from sub-Saharan Africa"
   Remove: Capitalization of "TransAtlantic"

   Response: Since there is no strong evidence in this study to draw this conclusion, the sentences have been modified to "**Phylogenetic analysis of 194 nucleotide positions showed no differences in the samples collected. The similarities suggests that there could be one species in this area. However, more robust studies with larger sample sizes are required to draw such conclusions. We also observed a clustering of the samples from Ghana with reference sequences from Africa and Brazil, suggesting they could be related. This study draws further attention to a neglected infection, presents the first characterisation of M. perstans in Ghana and calls for more population-based studies across different geographical zones to ascertain species variations and disease distribution**". Lines 34-40.

Introduction:

7. Change: some part -> parts (line 63)

   Response: "some part" has been changed to **"parts".**

8. Rephrase: lines 67 and 73, Consider combining sentences or using a different structure to avoid repetition about M. perstans being in Africa.

   Response: The paragraph has been modified accordingly.

9. Define: line 84, MDA (mass drug administration) on first use.

   Response: The MDA has been written in full on line 85

10. Italicize: line 88, Wolbachia

    Response: This has been done on line 89.

11. Remove: Comma after "age" in line 103

    Response: the comma has been removed on line 106

12. Lowercase: "Mansonellosis" unless it starts a sentence.

    Response: This has been corrected on line 111 and 114.

Methods & Results:

13. Clarify: Line 202, "Collared" might be unclear. Consider changing to "collected" for better understanding.

    Response: This has been corrected on line 158

14. Discussion Point: Lines 316-318 may be more appropriate for the discussion?

    Discussion:

15. Rephrase: Lines 396-402 can be rephrased to avoid repetition.

    Response: These lines have been reviewed

16. Change: line 409, suggest clarifying "supports work" -> "supports findings"

    Response: This section has been reviewed and now reads "**The clustering of the Ghana samples and other sequences from Gabon and Sierra Leone with the M. perstans ITS-1 (LT623911.1) from Brazil Amazona State (Fig 4) and other countries in Africa provides evidence of association. supports Findings work by of Tavares da Silva and colleagues (25) that also showed that a recently published genome of M. perstans from Brazil shared similarity with those from Africa."**
    Italicize: M. perstans (line 420)

17. Change: Vola -> Volta (line 431)

    Response: This has been corrected on line 355.

18. Additional Point: Briefly mention the possibility of low prevalence due to past ivermectin distribution programs in Ghana. (paragraph starting at line 427).

    Response: Possibility of IVM contributing to low prevalence and the consequences of stopping IVM treatment in these areas has been incorporated on lines 344-346.

19. Clarify: 6. I agree with the authors there should be a more comprehensive guide on the management and surveillance of M. perstans infections. However, in lines 443-450, it is not clear how low levels of M. perstans are alarming (as the prevalence found was low, albeit the limited sample size). Only if assumed that the ivermectin delivery in the past had a substantial impact on the prevalence of the parasite and, with onchocerciasis and LF elimination, ivermectin interruption could lead to the reestablishment of more M. perstans infections. Moreover, the link with malaria is not clear, despite the possible immunomodulation (as other filariae), which could make the host more susceptible to infectious diseases overall (potentially including malaria).

    Response: This portion has been reviewed and emphasis is now on the possibility of resurgence should IVM be discontinued in this area. Lines 343-347. Also the impact on malaria infections have been clarified on lines 360-373

    Overall, a brief revision for punctuation would benefit the study.

Editorial Comments

While revising your submission, please upload your figure files to the Preflight Analysis and Conversion Engine (PACE) digital diagnostic tool, https://pacev2.apexcovantage.com/. PACE helps ensure that figures meet PLOS requirements. To use PACE, you must first register as a user. Registration is free. Then, login and navigate to the UPLOAD tab, where you will find detailed instructions on how to use the tool. If you encounter any issues or have any questions when using PACE, please email PLOS at figures@plos.org. Please note that Supporting Information files do not need this step.

Response: The figures were uploaded to PACE before uploading them to the submission portal. The raw uncropped gel picture has been uploaded as Fig2_raw

If applicable, we recommend that you deposit your laboratory protocols in protocols.io to enhance the reproducibility of your results. Protocols.io assigns your protocol its own identifier (DOI) so that it can be cited independently in the future. For instructions see: https://journals.plos.org/plosone/s/submission-guidelines#loc-laboratory-protocols. Additionally, PLOS ONE offers an option for publishing peer-reviewed Lab Protocol articles, which describe protocols hosted on protocols.io. Read more information on sharing protocols at https://plos.org/protocols?utm_medium=editorial-email&utm_source=authorletters&utm_campaign=protocols.

Response: The laboratory protocols have now been deposited to protocls.io and available at http://dx.doi.org/10.17504/protocols.io.x54v928b4l3e/v1. This has been referred to in the method section on lines 213 and 226.

Journal Requirements:

Please review your reference list to ensure that it is complete and correct. If you have cited papers that have been retracted, please include the rationale for doing so in the manuscript text, or remove these references and replace them with relevant current references. Any changes to the reference list should be mentioned in the rebuttal letter that accompanies your revised manuscript. If you need to cite a retracted article, indicate the article's retracted status in the References list and also include a citation and full reference for the retraction notice.

Response: The references have been checked for completeness and is no retracted publication.

4 new references have been included.

a. Portela CS, Mendes de Araújo CP, Moura Sousa P, Gomes Simão CL, Silva de Oliveira JC, Crainey JL.Filarial disease in the Brazilian Amazon and emerging opportunities for treatment and control.Curr Res Parasitol Vector Borne Dis. 2023 Dec 23;5:100168. doi: 10.1016/j.crpvbd.2023.100168. eCollection 2024.
b. Macfie, J. W. S., & Corson, J. F. (1922). A new species of filarial larva found in the skin of natives in the Gold Coast. Annals of Tropical Medicine & Parasitology, 16(4), 465-471.
c. Agbemafle E, Kubio C, Ameme DK, Kenu E, Sackey S, Afari E. Evaluation of malaria surveillance system, Adaklu District, Volta Region, Ghana, 2019. International Journal of Infectious Diseases. 2020;101:412.
d. Ejigu BA, Wencheko E. Spatial Prevalence and Determinants of Malaria among under-five Children in Ghana. 2021.)

The list of key words has been updated to include, phylogenetic tree and Volta Region

Yours sincerely,

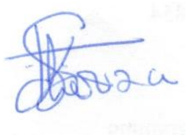

Prof. Dziedzom K. de Souza

On behalf of the authors
