# Supplementary material for: Identification and characterisation of Mansonella perstans in the Volta Region of Ghana
Source: PLoS One. 2024 Jun 7;19(6):e0295089. doi: 10.1371/journal.pone.0295089 (PMC11161070; doi:10.1371/journal.pone.0295089)
Supplement: Attachment — Submitted filename: Response to Comments.docx [file pone.0295089.s005.docx]

POINT BY POINT RESPONSE TO COMMENTS

# EDITORIAL COMMENTS

1. Some files have been removed from the portal as instructed.
2. Raw gel picture has been uploaded and labelled as fig2_ raw file.
3. Your ethics statement should only appear in the Methods section of your manuscript. If your ethics statement is written in any section besides the Methods, please move it to the Methods section and delete it from any other section. Please ensure that your ethics statement is included in your manuscript, as the ethics statement entered into the online submission form will not be published alongside your manuscript.

RESPONSE: The ethics statement has been moved to the methods section (134-142).

4. We note that Figure 1 in your submission contain [map/satellite] images which may be copyrighted. All PLOS content is published under the Creative Commons Attribution License (CC BY 4.0), which means that the manuscript, images, and Supporting Information files will be freely available online, and any third party is permitted to access, download, copy, distribute, and use these materials in any way, even commercially, with proper attribution. For these reasons, we cannot publish previously copyrighted maps or satellite images created using proprietary data, such as Google software

(Google Maps, Street View, and Earth). For more information, see our copyright guidelines: [http://journals.plos.org/plosone/s/licenses-and-copyright.](http://journals.plos.org/plosone/s/licenses-and-copyright)

RESPONSE:

a. The map has been revised and does not include any satellite or copyrighted images (Fig 1).
b. A description of how and data used to create the maps have been incorporated into the methods section for Fig 1. The maps were generated with an open-source QGIS software and the source of the shape files have been correctly referenced.
c. A screenshot of the copyright and email correspondence for free data usage have been uploaded as other.
d. Copyright information on the use of the shape files are as follows: *Creative Commons Attribution for Intergovernmental Organisations (CC BY-IGO)Under the CC BY-IGO license, you are free to share (copy and redistribute the material in any medium or format) and or adapt (remix, transform, and build upon the material) for any purpose, even commercially. The licensor cannot revoke these freedoms as long as you follow the license terms. The license terms are that you must give appropriate credit, provide a link to the license, and indicate if changes were made. You may do so in any reasonable manner, but not in any way that suggests the licensor endorses you or your use. Additionally, you may not apply legal terms or technological measures that legally restrict others from doing anything the license permits. When the Licensor is an intergovernmental organization, disputes will be resolved by mediation and arbitration unless otherwise agreed.*

**Reviewer #1:**

Introduction:

1. Your introduction comprehensively covers the species, distribution, transmission, clinical manifestations, and the main diagnostic methods of 'Mansonella perstans'. However, it could benefit from a clearer exposition on the current understanding (or lack thereof) of effective treatments for 'M. perstans', drawing a comparison with treatments available for onchocerciasis or lymphatic filariasis (LF) where at least one effective treatment is avaliable.

RESPONSE: The treatment for *M. perstans* in comparison to the other filarial parasites has now been described in lines 80-84.

2. The third paragraph, while informative, could be restructured for better flow and clarity. RESPONSE: The this has been addressed

3. Is there any historical data on 'Mansonella' in Ghana before? Understanding if there was a difference in the epidemiology of the parasite before and after (now) interventions for onchocerciasis and LF (as well as population growth and associated consequences) could be insightful

RESPONSE: Unfortunately, there haven’t been any studies to evaluate the impact of onchocerciasis and LF treatments on Mansonella in Ghana, hence we are unable to provide more information on this.

Methodology:

4. (2.1 Study Site) Figure A could be more well defined, although it is readable. I could only find the Hohoe and Adaklu districts, but not East Akyium district, understandably because the latter was negative for M. perstans; however, Figure 1 should not mention that M. perstans is only present in the two former districts, as it is part of the results, not the methodology.

RESPONSE: This has been corrected. The map has also been revised to include the other districts that were included in the bigger study.

5. (2.3 Sample Processing and Analysis) Please provide clarity on which filarial parasites were screened using Nested PCR (O. volvulus, M. perstans and M. perstans?) and the CO1 gene. Also, are these results presented, even if negative, in the result section?

RESPONSE: The Nested PCR assay was used, and this amplifies all the filarial parasites. However, only samples that were positive for M. perstans microscopy were screened. Notwithstanding, if all the samples were tested by PCR, there could have been more positives. Due to limited resources PCR wasn’t done on all the samples. This has been included as a limitation in the discussion section in lines All analysis were based on samples that tested positive for the ITS-1 gene. CO1 gene has been removed from the manuscript because, all the samples submitted were poorly sequenced. Lines 30, 48 and 178

6. (2.6 Sequence Analysis) The rationale for using the 'Loa Loa' sequence instead of a 'Mansonella perstans' sequence would benefit from explanation. If the choice was due to the lack of high-quality reference sequences from 'M. perstans', this should be explicitly stated.

RESPONSE: Loa loa was used as an outgroup due to low quality references and to allow for ingroup comparison. The write up has been updated to include this reason in lines 282 - 284

Results:

7. The range of microfilariae counts is indeed remarkable and adds significant value to the study. It would be beneficial to include the number of individuals screened for 'M. perstans' in East Akyim District, even if the results were negative.

1074 participants were tested in the East Akyim district and this has been included in the Methods section on line 241.

8. Additionally, the placement of each figure legend immediately following its corresponding description would aid in readability and comprehension instead of all at the end of the Results section.

RESPONSE: The figures have now been repositioned to appear after the just below their description.

Discussion:

9. The first paragraph of the Discussion section might be more appropriate in the Introduction, considering its content.

RESPONSE: The first paragraph of the discussion has now been removed and incorporated in the Introduction.

10. The phylogenetic analysis suggesting no variation between the non-contiguous Hohoe and Adalku districts raises intriguing questions about the potential prevalence of 'M. perstans' in surrounding districts. This could be an interesting avenue for future research to understand the prevalence of the parasite in the Volta region. Moreover, the discussion could be expanded to explore the possibilities of integrating 'M. perstans' surveillance with existing lymphatic filariasis and onchocerciasis programmes in Ghana. It would also be beneficial to discuss the broader public health implications of M. perstans, especially in relation to co-infections with other filarial diseases.

RESPONSE: The discussion has been updated to include the impact of the lack of variations in sequences on transmission in the surrounding districts as well as co-infections with other filarial parasites and the opportunities for integrating M. perstans studies in existing LF/onchocerciasis surveillance programs in the districts (lines 349-354).

**Reviewer #2:**

Opoku and Souza analysed M. perstans prevalence in the Volta region of Ghana and revealed low (up to 2.51%) infection rates. Moreover, they sequenced the samples and showed that the samples cluster with the reference sequences from other African countries and Brazil. It is very important to draw attention to this neglected filarial infection but the presented study lacks novelty and intense discussion (e.g., genome analysis). Moreover, the introduction and discussion lacks recent publication and is sometimes misleading. In my opinion, the manuscript need to be carefully re-written and updated before

RESPONSE: Most the sections have been updated and rearranged to help with articulation.

Major comments:

1) Line 80-82: The authors highlight that pathogenicity is not known/was so far not assessed, but the presented data sets do also not tackle this issue. Similar to Debrah et al. the presented manuscript shows the prevalence of M. perstans in a specific region of Ghana. In summary, the infection rates are very low compared to the middle belt of Ghana. The authors need to discuss that fact also in regards to the characteristics of the environment that are needed for M. perstans. For example Wanji and colleagues from Cameroon presented over the last years several publications about that.

RESPONSE: The introduction section has been updated to include prevalence from Debrah et al.,

2017 and Philips et al in the middle belt (lines 90-95). The discussion has been updated with reference to environmental characteristics and comparison made with the study areas in this study (lines 331-338).

2) A long this line, the introduction need to be updated, such as Mansonella spp. DEUX is not mentioned (line 49-50), the transmitting vector for M. perstans (C. milnei) have been deciphered in Cameroon (line 50-51), and it should be mentioned that M. perstans has no distinct clinical symptoms and is mostly asymptomatic (line 55-57).

RESPONSE: These have been included in lines 315, 66-68 and 103 respectively.

3) Line 64-67: Of course, mansonellosis is still neglected but the are several studies targeting Mansonella and it cannot be stated that it is only assessed during lymphatic filariasis or onchocerciasis surveillance programmes. Again, Wanji and colleagues specifically target Mansonella epidemiology, immunology and treatment efficacy (showing that ivermectin is not effective - should be included in line 68-70).

RESPONSE: This has been addressed and the introduction has been updated indicating ivermectin is not effective in lines 105, 84)

4) In my opinion, it is important to explain the applied methods more in detail. For example, the used PCR need to be shortly explained instead of only revising to a publication. What can be differentiated with this PCR (specificity/what filarial spp can be discriminated?). A long this line, Fig S1 shows a gel blot but there is no further explanation, such as what are the different bands in the sample lines. RESPONSE: The DNA and PCR methods have been explained in lines 160 – 215.

S1 has been changed to Fig 2 and now included in the results section and explained (lines 246, 251).

5) In regards to 4), supplement figures/tables need to be embedded, explained and in order within the text.

RESPONSE: This has been addressed accordingly.

6) How were the 30 samples that were sequences selected from the 39 positive individuals.

RESPONSE: Although there were 39 microscopy positive individuals, only samples that were positive by PCR were sequenced. This has been clarified in the text in line 213

7) line 229-230: There are several studies showing the impact of M. perstans on concomitant infections (Muhangi et al. 2007; Hillier et al. 2008; Stensgaard et al. 2016), which can be explained by the strong immune modulation of M. perstans (Ritter et al. 2018).

RESPONSE: This portion has been updated and the appropriate references inserted.

8) Recently, there are three publications about the genome of Mansonella, which need to be discussed extensively to place the presented results into the context.

RESPONSE: Recently published sequences have been mentioned and discussed.

**The line numbers indicated are in reference to the Revised Clean version document.
